# Delivery of Neuroregenerative Proteins to the Brain for Treatments of Neurodegenerative Brain Diseases

**DOI:** 10.3390/life14111456

**Published:** 2024-11-10

**Authors:** Eric T. Ebert, Kelly M. Schwinghamer, Teruna J. Siahaan

**Affiliations:** Department of Pharmaceutical Chemistry, School of Pharmacy, The University of Kansas, 2095 Constant Avenue, Lawrence, KS 66047, USA; eric.ebert@ku.edu (E.T.E.); kelly.schwinghamer@merck.com (K.M.S.)

**Keywords:** BDNF, NGF, IGF-1, LIF, neurotrophic factors, CNS, brain diseases, Alzheimer’s disease, multiple sclerosis, Parkinson’s disease

## Abstract

Neurodegenerative brain diseases such as Alzheimer’s disease (AD), multiple sclerosis (MS), and Parkinson’s disease (PD) are difficult to treat. Unfortunately, many therapeutic agents for neurodegenerative disease only halt the progression of these diseases and do not reverse neuronal damage. There is a demand for finding solutions to reverse neuronal damage in the central nervous system (CNS) of patients with neurodegenerative brain diseases. Therefore, the purpose of this review is to discuss the potential for therapeutic agents like specific neurotrophic and growth factors in promoting CNS neuroregeneration in brain diseases. We discuss how BDNF, NGF, IGF-1, and LIF could potentially be used for the treatment of brain diseases. The molecule’s different mechanisms of action in stimulating neuroregeneration and methods to analyze their efficacy are described. Methods that can be utilized to deliver these proteins to the brain are also discussed.

## 1. Introduction

Neurodegenerative diseases represent a wide variety of conditions that affect many people worldwide. The number of people who have Alzheimer’s disease (AD) in the U.S. and worldwide are 6.7 and 55 million, respectively [1]. For Parkinson’s disease (PD), there are about 500 thousand and 10 million patients in the U.S. and worldwide, respectively [2]. There are 2.8 million people who have multiple sclerosis (MS) worldwide, with about 1 million MS patients in the U.S. [3]. Many brain diseases do not have viable treatment options that reverse the effects of the disease; most treatments only treat the disease symptoms and do not address neurodegeneration problems in the central nervous systems (CNS). Current research efforts for treating brain diseases focus on designing various therapeutic modalities for CNS that can prevent neurodegeneration or stimulate neuroregeneration. These potential therapeutic agents include (a) gene therapy vectors; (b) proteins (i.e., hormones, antibodies, and enzymes); (c) peptides; and (d) small molecules [4,5,6,7,8,9,10,11,12,13,14]. To be effective, these agents should be delivered to the CNS. For neuroregeneration, neurotrophic factors such as brain-derived neurotrophic factor (BDNF), nerve growth factor (NGF), and peptides/peptidomimetics growth factors have been investigated to reverse AD, PD, and MS (for an excellent review on the delivery of neurotrophic factors to the brain, see Bahlakeh et al., 2021) [15]. Treatment with growth and neurotrophic factors to induce neuroregeneration in the CNS could reverse these diseases by restoring the natural function of neurons. Thus, we would like to discuss the progress that has been made in treating neurodegenerative diseases using molecules that promote neuroregeneration. This review focuses on neurotrophic factors and a cytokine that have been investigated to treat brain diseases such as AD, PD, and MS. These molecules include BDNF, NGF, insulin-like growth factor-1 (IGF-1), and leukemia-inhibitory factor (LIF). Their mechanisms of action are discussed. We also discuss several methods that have been developed to overcome the many challenges for delivering neurotrophic factors to the brain.

The blood–brain barrier (BBB) is the ultimate barrier that prevents therapeutics from crossing into the brain. To induce the neuroregenerative effects, the growth and neurotrophic factors must reach the brain. Unfortunately, many neurotrophic factors cannot readily cross the BBB and thus cannot enter the brain from systemic circulation [4,16,17]. Many therapeutic proteins (i.e., hormones, antibodies, and enzymes) also fail to treat various brain diseases because they cannot cross the BBB. The physicochemical properties (i.e., size, hydrogen bonding potential, and charge) of these molecules are not conducive to passively diffuse across the BBB via either the transcellular (partitioning through cells) or paracellular (diffusing between cells) pathways [18]. Efflux transporters such as P-glycoproteins (Pgp) and multidrug resistant proteins (MRP) on the surface of the BBB can prevent small molecules from passing through the transcellular pathway [19,20,21]. In contrast, nutrient transporters on the BBB (i.e., amino acid, peptide, and glucose transporters) are present to ensure essential nutrients enter the brain [22]. These transporters have been exploited for delivering small-molecule therapeutic agents to the brain. Similarly, some select proteins have transport receptors, so they can pass through the BBB via receptor-mediated endocytosis process.

Besides various methods for delivering neurotrophic factors to the brain, there are other factors that need to be considered for successfully utilizing them to treat neurodegenerative diseases. One of the factors is their kidney clearance, as the molecular weight of most neurotrophic factors is less than 65 kDa; therefore, they have a fast renal clearance due to glomerular filtration [23]. The fast clearance of neurotrophic factors leads to a short residence time in the systemic circulation, creating a short window for their deposition and distribution in the brain [23]. In addition, the plasma half-life or plasma stability of neurotrophic factors should also be considered when developing neurotrophic factors as therapeutics. Therefore, the pharmacokinetic and pharmacodynamic profiles of neurotrophic factors become important considerations for developing them into therapeutics. All of these factors contribute to the effectiveness and efficiency of delivering neurotrophic factors to the brain. Getting through the BBB is just the first hurdle for neurotrophic factors to become effective therapeutics. After crossing the BBB, these molecules need to diffuse through brain tissues to the site of action in the brain. Although neurotrophic factors can be delivered to the brain, their clearance from the brain through cerebral spinal fluid (CSF) should also be studied [24,25]. Thus, brain retention and distribution of neurotrophic factors at the target active site in the brain are important for their effectiveness as therapeutic agents [25].

## 2. Methods for Delivering Neurotrophic Factors to the Brain

Methods such as focused ultrasound with microbubbles (FUS-MB), hypertonic mannitol, receptor-mediated transcytosis, peptide-mediated BBB modulation, and intranasal delivery methods have been investigated to deliver neurotrophic factors to the brain [26]; however, some of them have not gone beyond the preclinical stage and need validation for use in humans (for an excellent review, see Whelan et al., 2021 [27]). In addition, the safety features of many of the previously mentioned methods need to be investigated to ensure that multiple treatments can be given with no adverse effects. Invasive techniques such as intracerebroventricular (ICV) injection can deliver neurotrophic factors directly to the brain by injecting it directly into the cerebral ventricles of the brain. Alternatively, implants containing the therapeutic molecule can be directly inserted into the brain [16]. These invasive methods are inconvenient and potentially dangerous to patients because they require surgery that may cause brain infection [16]. Currently, ICV administration has been utilized to deliver antibiotics and recombinant enzymes in humans [26,28,29,30,31].

Many different non-invasive methods have been investigated for improving the delivery of molecules across the BBB via the transcellular pathway [27]. The receptor-mediated transcytosis strategy involves delivering molecules through the transcellular pathway by allowing the therapeutic molecules to bind to receptors, followed by receptor-mediated endocytosis into the capillary endothelial cells [32]. The molecules are then transported through the endothelial cells and released into the parenchyma. For example, IGF-1 can cross the BBB using the IGF-1 receptor (IGF-1R) [33]. Alternatively, neurotrophic factors can be delivered across the BBB endothelial cells by conjugating it to the ligand of the transcytosis receptor [16]. For example, BDNF conjugated to an antibody that binds to transferrin receptor on the BBB endothelial cells can deliver BDNF across the BBB [34]. Some nanoparticles have been decorated with antibodies that recognize the transferrin receptor for delivering them across the BBB [35].

Another method to deliver neurotrophic factors and other proteins to the brain is via paracellular pathways of the BBB. Due to the low porosity of the tight junction of the BBB, the intercellular junctions must be disrupted to allow large proteins such as neurotrophic factors to penetrate through the BBB. Several potential methods include osmotic brain delivery, focused ultrasound, and the *adherens* junction BBB modulation approaches. All these methods disrupt the intercellular junction cell–cell adhesion to enhance the porosity of the paracellular pathways [16].

For osmotic delivery method, hyperosmotic mannitol along with the therapeutic agent are infused through the carotid artery [24,36]. The hypertonic mannitol transiently opens the paracellular pathway by shrinking brain endothelial cells, thus allowing the therapeutic molecule to diffuse into the brain [24,36]. Clinically, hypertonic mannitol has been utilized to deliver both small-molecule and antibody therapeutics to glioblastoma patients [37,38,39].

FUS-MB can mechanically disrupt the BBB intercellular junction, allowing molecules to diffuse across the BBB into the brain [40,41]. This method has been used both preclinically and clinically to deliver therapeutic molecules to the brain [42,43,44].

Cadherin peptides, called BBB modulators (BBBMs), disrupt cadherin–cadherin interactions in the *adherens* junction of the BBB to transiently increase the porosity of the BBB paracellular pathway, allowing the diffusion of therapeutic molecules into the brain [4,17,45]. In this case, BBBMs can enhance the delivery of small to large molecules, including monoclonal antibodies, into the brain [24,25,45,46,47,48,49,50,51,52,53,54].

Finally, intranasal drug delivery has been developed to deliver therapeutics into the olfactory part of the brain by avoiding the BBB [55,56,57,58,59,60,61]. Intranasal delivery has also been combined with FUS-MB to deliver molecules to the brain [62,63]. Multiple pathways exist for the drug to enter the brain from the nasal cavity, but some mechanisms involve transport along olfactory nerves [4,55].

Overall, there are many methods that have been used to deliver molecules to the brain. Many of these methods have been used in preclinical models of neurodegenerative diseases; however, some methods, such as FUS-MB, have reached clinical use. Many of the methods have slightly different mechanisms of action to disrupt the BBB. For many of the methods, the safety associated with repeated treatments needs to be assessed. Repeated disruption of the BBB could result in damage to the brain vasculature. Therefore, the safety profiles of these methods should be investigated and established.

## 3. Potential Treatments of Alzheimer’s Disease

AD is a neurodegenerative disease that includes symptoms such as memory loss and declining cognitive ability [64]. These disease symptoms are related to neurodegeneration in the brain as the disease progresses [64]. The amyloid cascade hypothesis has been proposed for the etiology of AD in which the formation of amyloid-β (Aβ) plaques occur before the generation of intracellular tau neurofibrillary tangles (NFTs); this is due to hyperphosphorylation of intracellular tau protein, which causes neuronal death [65]. The activity of β- and γ-secretases in cleaving amyloid-precursor protein (APP) produces the accumulation of amyloid beta peptides, thus causing amyloid plaque formation [66]. Amyloid plaques cause neuronal toxicity through various mechanisms such as oxidative stress or activating neuronal receptors [66]. In the case of tau, hyperphosphorylation of tau decreases its ability to bind to microtubules and leads to aggregation and neuronal toxicity [67]. It is believed that though Aβ is related to the onset of AD, the severity of the disease is correlate to tau pathology, which is supported by studies that show reduced Aβ toxicity in tau-knockout mice [68]. The prion hypothesis suggests that tau aggregates escape their original cells and enter adjacent cells to further seed tau aggregation and cause the disease to spread [69]. Current strategies to treat AD are aimed at removing Aβ peptides from the systemic circulation that presumably impact the Aβ plaque formation. Aduhelm^®^ (aducanumab, Biogen Inc., Cambridge, MA, USA) and Leqembi^®^ (lecanemab, Eisai Co, Tokyo, Japan and Biogen Inc., Cambridge, MA, USA) have been shown to reduce amyloid-β plaques in early disease stage of AD patients [5,70].

Vaccines have shown promising results in animal model of AD as well as in AD patients; the goal is to generate antibodies to reduce Aβ or tau oligomers [6]. Active vaccination using human aggregated Aβ1–42 (AN1792) along with QS-21 adjuvant reduced plaque loads when analyzed postmortem; however, it did not slow down dementia progression in patients [6]. Unfortunately, a small patient population suffered from meningoencephalitis during clinical trials of this vaccine [6].

The failure of many anti-Aβ immunotherapies in clinical trials has led many researchers to shift their focus on targeting tau as a potential treatment for AD. Active immunization strategies have also proven to be successful in reducing tau pathology [7,8]. Anti-tau antibodies prevent the spread of tau pathology by blocking the aggregated tau fibril’s access to adjacent cells. Passive anti-tau immunization improved cognitive performance, reduced tau pathology, and delayed onset of motor function decline in mouse models of AD [7,8]. Anti-tau mAb, Semorinemab, showed potential to improve outcomes of patients with prodromal or mild AD; however, it failed during clinical trials, causing skepticism among scientists on the passive immunization strategy for the treatment of AD [9,10]. There are several explanations as to why Semorinemab failed in clinical trials. First, the mAb may have targeted the wrong tau epitope. Second, the clinical trial was focused on the wrong patient population. Third, the injected mAb may act as a foreign substance that is cleared by the immune system. Nonetheless, many different anti-tau mAbs are being developed and undergoing clinical trials with the intention of delaying or preventing neurodegeneration in AD patients. The AADvac1 vaccine was developed using a selected tau domain from the tau–tau interaction region to induce the production of IgG mAb against pathologic tau, and it was successful in phase I clinical trials [13,14,71,72]. In a phase II study, AADvac1 induced a robust immune response in 98.2% of patients and slowed down the increase in neurofilament light chain (Nfl) as a marker for neurodegeneration [11]. Overall, the current treatment strategies for AD only help with symptoms and do not reverse the progression of the disease. Many of the treatment strategies attempting to remove Aβ plaques or tau aggregates have limited success. Thus, neuroregenerative therapies have been investigated as an alternative strategy to reverse AD progression.

## 4. Neurotrophin Signaling Pathways

**Figure 1:**
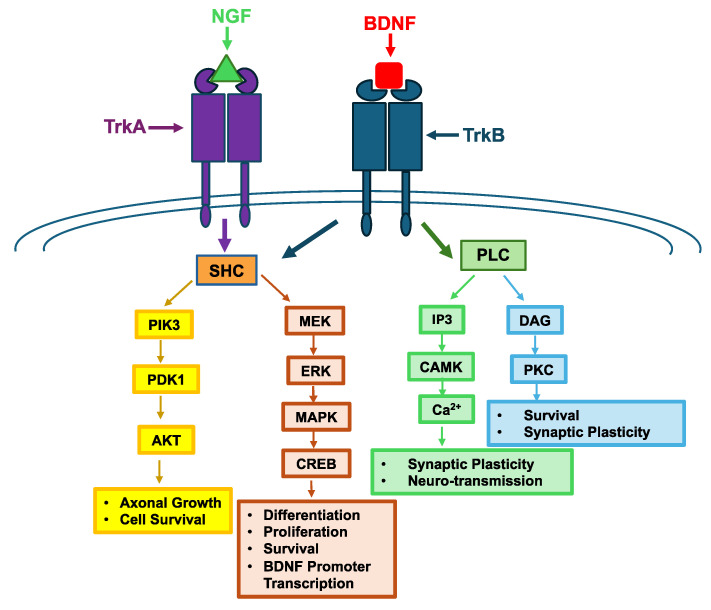
The effects of BDNF and NGF on the cell signaling processes that potentially lead to cellular effects with the end results of neuroregeneration or neurorepair. BDNF binds to a homodimeric form of TrkB receptor, and NGF binds to a homodimeric form of TrkA receptors on the cell surface. Upon binding of BDNF and NGF to TrkB and TrkA, respectively, they stimulate SHC pathway activation to activate PIK3, followed by activation of PDK1 and Akt signaling pathways to generate axonal growth and cell survival. The second SHC pathway is via MEK that activates ERK and MAPK, followed by activation CREB to generate cell differentiation, proliferation, survival, and BDNF promotor transcription. BDNF activates the PLC pathway, followed by activation of IP3 and CAMK signals for generating synaptic plasticity and neurotransmission. The PLC pathway also activates DAG and PKC signals for cell survival and synaptic plasticity. Abbreviations: NGF, nerve growth factor; BDNF, brain-derived neurotrophic factor; TrkA, tropomyosin related kinase A; TrkB, tropomyosin-related kinase B; SHC, Src homology and collagen; PI3K, phosphatidylinositol 3-kinase; PDK1, 3′-phosphoinosotide-dependent kinase-1; AKT, protein kinase B; MEK, mitogen-activated protein kinase kinase; MAPK, mitogen-activated protein kinase; CREB, cAMP response element-binding protein; IP3, inositol triphosphate; CAMK, Ca^2+^/calmodulin-dependent protein kinase; DAG, diacylglycerol; PKC, protein kinase C.

One of the most well-known families of neurotrophic factors is the neurotrophins, whose members include BDNF, NGF, neurotrophin 3, and neurotrophin 4/5 [73]. Mature neurotrophins are noncovalently associated homodimers that are synthesized as precursor proteins [74]. To exert their effects, neurotrophins bind to tropomyosin-,related kinase receptors (Trk) and the p75 neurotrophin receptor (p75^NTR^) [75]. Different neurotrophins bind to different subtypes of Trks. For example, TrkB binds to BDNF and neurotrophin 4/5 with high affinity while TrkA binds to NGF with high affinity (Figure 1) [75].

The evidence suggests that both Trk and p75^NTR^ receptors need to be present to create a high-affinity binding site for the neurotrophin and induce neurotrophic effects [76]. BDNF can bind to TrkB without p75^NTR^, but it needs p75^NTR^ to activate certain signaling pathways [77]. Interestingly, the p75^NTR^ receptor has its own activity when it binds to a neurotrophin, and the activity also depends on its binding to other co-receptors during neurotrophin binding. In the absence of Trk receptors, p75^NTR^ can initiate apoptosis in neuronal cells; therefore, the effect of neurotrophins on neuronal cells can be dependent on what receptors are most available for the neurotrophin to bind [78]. In the case of NGF and TrkA, cells internalize NGF at a faster rate when TrkA and p75^NTR^ are present [79]. TrkA phosphorylation and activation by NGF is enhanced when p75^NTR^ is present [80]. p75^NTR^-deficient dorsal root ganglion cells were less sensitive to NGF and needed more NGF to survive. In summary, both the Trk and p75^NTR^ receptors are needed to induce a robust response from the neurotrophic factor [81].

Multiple pathways can be activated upon neurotrophin binding to the Trk and p75^NTR^, which include Ras activation, leading to downstream signaling through the mitogen-activated protein kinase pathway, the phospholipase C-γ1 (PLC) pathway, and the phosphatidylinositol 3-kinase (PIK3) pathways (Figure 1) [75]. For the signal transduction event, the phosphorylated Trk binds to Src homology and collagen (SHC), which is then itself phosphorylated [82]. Various other adaptor proteins bind to the complex, resulting in activated Ras and leading to the mitogen-activated protein kinase pathway or the activation of PIK3, leading to the activation of its pathway [82]. The activation of these pathways ultimately changes gene transcription through the activation of cAMP-response element binding protein (CREB). As a result, gene expression changes, ultimately influencing neuronal survival. In addition to this pathway, TrkB can also activate PLC, which can lead to an influx of calcium ions into the neuron, thus increasing synaptic plasticity [83]. Ultimately, the changes caused by neurotrophic factor signaling lead to an increase in neuronal survival, proliferation, and plasticity.

Neurotrophin effects could be limited to various brain areas because some Trk receptors are expressed at different levels in different brain regions. For example, TrkA was detected in multiple brain regions using immunohistochemical staining; however, TrkA only co-localized with p75^NTR^ in the basal forebrain [84]. Therefore, NGF would have significant neurotrophic activity in the basal forebrain where both TrkA and p75^NTR^ are expressed. Similarly, a high expression level of TrkB receptors can be found in the hippocampus and thalamus [85]. The effects of a neurotrophin in a specific brain area are further complicated by the fact that each neurotrophin can activate each Trk with varying affinities. For example, dopaminergic neurons do not express TrkA, but they express TrkB; interestingly, treatment of dopaminergic neurons with BDNF and NGF stimulated the release of dopamine [86]. However, a much higher dose of NGF was needed to stimulate the dopamine release. Similarly, BDNF has been demonstrated to provide protection to neurons in the basal forebrain, suggesting that either the TrkB receptor must be expressed in the basal forebrain, or it may activate the TrkA receptor to a certain degree. Therefore, the appropriate neurotrophin should be delivered to a specific brain area to have its maximal effect for treatment.

## 5. BDNF in Neurodegenerative Diseases

BDNF has significant roles for a healthy nervous system, where it promotes neurogenesis and synaptic plasticity [74]. BDNF regulates synaptic transmission and long-term potentiation in the hippocampus [87]. It was found that BDNF knockout mice died within a few days of birth, and mice heterozygous for the BDNF gene have learning defects [73]. The loss of both NGF and BDNF have been correlated with multiple neurodegenerative diseases [73].

The role of BDNF has been described in Huntington’s disease, where the transport of BDNF from the cerebral cortex to striatum was weakened; thus, BDNF may be delivered to the striatum for this disease. BDNF can reduce cell death in cells transfected with a huntingtin (HTT) mutant [74,88]. When mice with the HTT mutant were crossbred with heterozygous BDNF-knockout mice, the mice had earlier disease onset with more severe motor dysfunction than the parent mice, suggesting that BDNF has important functions in the brain. Clinical trials for using BDNF to treat amyotrophic lateral sclerosis (ALS) patients failed to improve patient conditions. ALS is characterized by the atrophy of motor neurons, leading to gradual loss of motor function [89,90]. This failure could be attributed to the ineffective delivery of BDNF to the patient’s brain region with degenerating neurons [89,90]. Treating wobbler mice (a model for ALS) with BDNF prevented corticospinal motor neuron degeneration, indicating the BDNF has efficacy in preventing neurodegeneration.

BDNF also has roles during stroke. Stroke patients had lower levels of BDNF compared to healthy controls, suggesting that these stroke patients may have reduced potential to recover from the stroke [22,34,90,91,92,93,94]. Patients who suffered from stroke normally had neuronal degeneration in the cerebral cortex. Blocking the activity of endogenous BDNF worsened the cerebral ischemia condition. Therefore, it was proposed that treatment with BDNF might improve patient’s conditions. Because stroke can cause leaky BBB, it was proposed that BDNF can penetrate the BBB through the leaky BBB paracellular pathways. Unfortunately, because the amount of BDNF that permeated through the BBB was low, a large dose of BDNF with repeated administrations were needed to treat stroke patients to achieve a sufficient dose in the cortex. The large dose made BDNF treatment impractical and could generate adverse side effects. The leaky BBB suggested to occur during stroke was not leaky enough to allow BDNF to pass into the brain. Therefore, BDNF may be useful for treating stroke patients but needs to be delivered to the brain and cannot rely on the leaky BBB for traveling into the brain.

### 5.1. BDNF in Alzheimer’s Disease

Delivery of BDNF into the brain has been investigated in animal models of AD [47,95,96,97]. The goal is to deliver BDNF to stimulate neuroregeneration and repair to restore brain function back to normal. Unfortunately, BDNF cannot readily cross the BBB due to its physicochemical properties; thus, achieving sufficient brain concentrations of BDNF becomes an obstacle for using it as a therapeutic. Normally, BDNF is widely distributed throughout the CNS [98]. In AD patients, BDNF levels were deficient or lower than normal in the entorhinal cortex and hippocampus [96]. This deficiency causes symptoms associated with AD because BDNF release is necessary for maintaining the electrophysical function of the memory circuitry. To study BDNF in AD, various mouse models have been developed. One mouse model of AD is the P301L model, which expresses mutated human Tau, leading to tauopathy [99]. It was found that AD patients and P301L mice have lower than normal BDNF levels in the serum and the brain [95]. Increasing BDNF expression can slow down cognitive decline in AD patients with advanced disease; therefore, BDNF levels have been used as a biomarker to help determine disease progression in AD patients [100,101]. The lower levels of BDNF associated with AD lead to neurodegeneration. Many of the neuroprotective signaling pathways would be lost due to a lack of BDNF (Figure 1) [74]. Tau protein can inhibit the production of BDNF, which could lead to further worsening of neurodegeneration [102]. In addition, Aβ can decrease BDNF mRNA levels in SY5Y cells, suggesting that accumulation of Aβ will also contribute to lower levels of BDNF [103]. Overall, the lack of BDNF’s neuroprotective signaling contributes to the progression of AD.

BDNF expression was shown to influence the pathology in APP overexpressing mice, but the amyloid plaque density did not change upon treatment with BDNF in APP mice, suggesting that BDNF works independent of the plaque formation [96,97]. Administration of BDNF-expressing cells in AD mice has been shown to improve disease outcomes for AD mice [96,97]. Delivery of the BDNF gene into the entorhinal cortex in aged rats reversed age-related memory changes, prevented cell death, and improved learning and memory, with no adverse side effects [96,97]. This concludes that BDNF treatment delays cell death of the entorhinal–hippocampal circuitry in AD [96,97].

### 5.2. Delivery of BDNF Across the BBB in AD and Other Brain Diseases

Recently, BDNF was delivered to the brain using a blood–brain barrier modulator (BBBM) called ADTC5 peptide (Cyclo(1,7)Ac-CDTPPVC-NH_2_) in the APP/presenilin-1 (APP/PS1) transgenic mouse model [47]. Treatment with ADTC5 and BDNF led to improved performance in the Y-maze test and the novel object recognition (NOR) test compared to BDNF alone [47]. BDNF treatments with ADTC5 enhanced the expression of NG2 receptors in glial cells as well as increasing EGR1 and ARC mRNA transcripts in the brain [47].

BDNF gene delivery has also been utilized in the treatment of various diseases. To stimulate the production of BDNF in the brain, the BDNF gene was delivered into the brain of transgenic APP mice using adeno-associated viruses (AAV) or AAV-BDNF [95]. In this case, a direct injection of the gene vector was used. The results showed an increase in BDNF production in the hypothalamus of treated mice. The AAV-BDNF-treated mice exhibited improvement in memory and learning capability [95]. In this study, delivering AAV-BDNF reversed cell and synapse damage, and it had therapeutic and protective effects against tauopathy and Aβ-related pathologies in AD [95,96,97]. In the entorhinal cortex and hippocampus, elevating the BDNF level caused neuronal recovery in non-human primates; thus, BDNF was proposed to have an important function in pathogenesis of AD [97]. AAV-BDNF did not influence tau phosphorylation but decreased some proinflammatory cytokines (i.e., IL-6, IFN-γ, TNF-α, and IL-1β). Upregulation of BDNF in serum and the brain increased the expression of postsynaptic density 95 (PSD-95), PSD-93, synapsin-1 (syn-1), NAP-25, and vesicle-associated membrane protein 1 (VAMP-1) proteins in the synapse and also improved behavior and learning in P301L mice [95,97].

Multiple strategies have been investigated to increase BDNF’s half-life in the systemic circulation as well as BBB penetration. Wu and Pardridge (1999) conjugated polyethylene glycol (PEG) to BDNF to increase its plasma half-life [104]. The PEGylated BDNF was also conjugated with one biotin molecule so that the OX26 mAb–streptavidin conjugate could also be attached. OX26 mAb increases penetration through the BBB by targeting the transferrin receptor to promote receptor-mediated endocytosis. Careful attention was used to place the biotin on the PEGylated BDNF so that the interaction with streptavidin did not interfere with either BDNF or OX26 interaction with their receptors [104]. Daily administration of the PEGylated BDNF/OX26 conjugate for 6 days after induction of forebrain ischemia helped prevent the loss of hippocampal neurons [104].

Various nanoparticles, liposomes, and other nanoscale formulations have also been utilized to help deliver BDNF or the BDNF gene to the brain. BDNF formulated with a block copolymer of PEG and poly-L-glutamate increased the brain penetration relative to free BDNF [93]. This formulation was able to improve the recovery of mice after stroke [93]. Liposomes studded with transferrin ligands that contain BDNF genetic material have been shown to successfully traverse through the BBB after IV administration [105]. In vitro, BDNF peptides on nanoparticles as well as in hydrogel can stimulate the TrkB receptor and promote axon regeneration of the sciatic nerve, improving the recovery rate in rats with sciatic nerve injury [106,107]. After stroke, mice were implanted with BDNF-containing hydrogel in the infarct cavity; the implanted BDNF hydrogel promoted neurogenesis and recovery from stroke [92]. BDNF-loaded nanoparticles made of poly(lactic-co-glycolic acid) (PLGA) coated with poloxamer 188 were able to enhance recovery in rats with traumatic brain injury [104].

Overall, many strategies have been utilized to deliver BDNF to the brain in AD. Many of these strategies have their advantages and disadvantages. In the case of gene delivery, off-target effects and how long the effects of the gene delivery last are concerns that will need to be addressed before its use in humans.

### 5.3. Intranasal (IN) Delivery in Brain Diseases

Instead of delivering molecules across the BBB, the intranasal (IN) route is another route of administration that allows molecules to access the brain by bypassing the BBB [108]. After administration into the nasal cavity, molecules travel in the nasal cavity to either the respiratory region containing trigeminal nerves or the olfactory region containing nerves [109]. In the olfactory region, molecules enter the olfactory bulb intra- or extracellularly through the olfactory sensory neurons [55]. From there, the molecules can travel to the rest of the brain through perivascular spaces [108]. Molecules can also pass through the paracellular gaps in the olfactory epithelium [55]. The trigeminal nerve pathway is similar to the olfactory nerve pathway, except that the molecules will be released in the pons and then distribute throughout the rest of the brain [55,109]. Studies have found that intranasally administered NGF molecules are distributed mostly to the olfactory bulb and to other areas of the brain at a lower concentration [56,57]. IN delivery of BDNF has been evaluated in various neurodegenerative disease models [58,59,62]. IN delivery of BDNF in AD11 mice improved their recognition memory; however, the treatment did not influence amyloid beta or tau pathology regardless of BDNF doses [58].

### 5.4. BDNF in Parkinson’s Disease (PD) and Traumatic Brain Injury

PD is the second most common neurodegenerative disease following AD. PD is due to the loss of dopaminergic neurons in the substantia nigra pars compacta [110]. Due to dopamine deficiency, there is an excessive inhibitory input to the thalamus that hinders signal transduction back to the cortex [110]. Normal signal transduction is necessary to regulate smooth, controlled movements. The effects of dopamine deficiency can be seen in the symptoms of PD, which include tremors, rigidity, bradykinesia, and postural instability. In addition to these clinical manifestations of PD, Lewy bodies, which are composed of α-synuclein (αS) proteins, are also observed in the brains of PD patients and can be used as a diagnostic marker for PD [110]. There is evidence to indicate that environmental and genetic factors can lead to abnormal forms of αS that contribute to the death of neurons; however, the precise cause of PD remains elusive [111]. New potential disease-modifying targets for developing treatment of PD are currently being identified from pathogenesis of PD.

The current standard for the treatment of PD includes medications such as levodopa (L-DOPA) to supplement the lack of dopaminergic neurons [112]. However, the long-term use of L-DOPA could have complications such as an increase in dyskinesia and the “on-off” effects that are observed [112]. Other strategies to increase dopamine in the brain include other dopamine receptor agonists or drugs to reduce the breakdown of dopamine, such as monoamine oxidase inhibitors [112]. Reducing the activity of acetylcholine and N-methyl-D-aspartate receptors is also a useful treatment option [112]. Other non-pharmaceutical treatment strategies include ablative surgery, deep brain stimulation, and physical therapy; however, ablative surgery and deep brain stimulation are invasive procedures that are not easily performed [112]. It is important to note that all of the currently approved treatments for PD do not cure the disease but only manage the symptoms.

BDNF has been investigated for treatment of Parkinson’s disease (PD) because a lower mRNA expression of BDNF was found in PD patients compared to healthy individuals [113]. In the PD animal models, brain delivery of BDNF prevents neuronal death at the striatum and substantia nigra, where the TrkB receptors are expressed by the dopaminergic neurons [86]. In vitro, BDNF induces secretion of dopamine in neurons and protects dopaminergic cells from induced toxicity by 1-methyl-4-phenyl-1,2,3,6-tetrahydropyridine (MPTP) [86,114]. Many strategies have also been used to deliver BDNF to the brains of PD mouse models. In vivo, FUS-MB has been utilized to deliver BDNF to the brain in PD. A combination of IN administration and FUS-MB enhanced BDNF penetration into the brain [62,63]. FUS-MB opens the paracellular junctions of the BBB and increases perivascular pumping action to allow penetration of BDNF into the brain parenchyma [62,63]. Interestingly, the amounts of BDNF in the brain hemisphere targeted with FUS were not significantly different than the non-targeted side [62]. In another study, the increase in delivery of BDNF to the brain was not statistically different compared to when BDNF was delivered intravenously with FUS [63].

### 5.5. BDNF in Multiple Sclerosis (MS)

MS is a neuroinflammatory disease of the CNS that results in large focal lesions in the white matter of the brain and spinal cord. It is widely believed that MS is caused by circulating leukocytes penetrating the BBB, moving into the CNS, and attacking and destroying the myelin sheath, which coats the neuron axon, causing disruption in signal transmission for neurons [115,116]. The loss of neuronal function in the brain and spinal cord can generate a range of neurological deficits, including sensory loss, limb weakness, partial vision loss, and other symptoms [116]. There are multiple forms of MS, including relapsing–remitting (the most common), secondary progressive, primary progressive, and progressive–relapsing [117]. The relapsing-remitting form of MS (RRMS) is characterized by periods of disease progression followed by periods of disease remission; this is the most common form of MS [118]. Other forms of MS involve continued progression of disease symptoms with no remission [117].

All current therapies for MS modify disease progression or symptoms. Many of the current drugs modulate the immune response to reduce disease progression. These approved therapies include interferon beta, glatiramer acetate, mitoxantrone, fingolimod, dimethyl fumarate, and monoclonal antibodies that target various components of the immune system [116,117]. A variety of new drugs, including small molecules, proteins, and cell-based therapies, have been investigated in clinical trials for MS with varying levels of success [119]. Mesenchymal stem cells (MSCs) showed efficacy in preclinical models of MS; they differentiated into oligodendrocytes and induced Treg cells’ proliferation [120]. In MS clinical trials, although MSCs were safe and well tolerated, the efficacy of MSC was inconclusive because of the contradictory results from different studies. The disadvantages of MSC therapies are (a) the potential of MSC stimulating tumorigenesis and (b) the limited numbers of MSC that arrive at the target site after a systemic administration. However, all of these therapeutics do not address the neurodegeneration that occurs during MS.

The lack of a therapeutic alternative to cure MS creates opportunities to develop drugs that not only treat the symptoms but reverse the disease by restoring the function of damaged neurons. None of the currently available treatments address neuroregeneration to repair the neuronal damage that is observed in MS; therefore, neurotrophic factors represent a promising class of drug that has potential to reverse MS. Especially in the progressive form of MS, where the neurodegeneration is very prominent, neurotrophic factors may have the potential to reverse the neurological damage caused by the disease. The ADTC5 peptide BBBM enhanced the delivery of BDNF to the brain in an experimental autoimmune encephalomyelitis (EAE) model of MS in mice [46]. Co-administration of ADTC5 and BDNF improved the clinical scores of mice relative to treating with PBS, BDNF alone, and ADTC5 alone [46]. The mice treated with BDNF + ADTC5 showed upregulation of the NG2 receptor as well as early growth response 1 (EGR1) and activity-related cytoskeleton-associated protein (ARC) mRNAs, indicating neuroregeneration. ADTC5 and other BBBM peptides are a promising solution for overcoming the poor brain penetration of neurotrophic factors. Cuprizone-fed mice treated with BDNF-containing exosomes had improved motor function and increased myelination relative to controls [121]. Mice with spinal cord injury showed a faster recovery when treated with BDNF mRNA encapsulated using a cationic polymer [122]. This suggests that delivering BDNF mRNA can stimulate BDNF production to induce neuroregeneration in mice with spinal cord injury. Overall, BDNF has great promise for the treatment of MS, especially to undo the neurodegeneration that is associated with advanced forms of the disease.

## 6. Nerve Growth Factor (NGF) in Neurodegenerative Brain Diseases

NGF has been implicated in the development and maintenance of the CNS, and it has an important role in the survival and function of cholinergic neurons [123]. Similar to BDNF, NGF exerts its effects through the TrkA and p75^NTR^ receptors, leading to the activation of multiple pathways for neuronal survival and regeneration [75]. NGF regulates memory, arousal, consciousness, and attention [123]. NGF can regulate the differentiation of immune cells [123]. A lethal effect was observed in NGF-knockout mice within a few days, and reducing NGF expression resulted in significant muscular defects in adult mice [124,125]. Overall, NGF is important for both the development and normal functioning of the nervous system. Given the importance of NGF in the CNS, many neurodegenerative diseases involve the absence or dysfunction of NGF.

### 6.1. NGF and Alzheimer’s Disease

The role of NGF in AD seems to be unclear because there are conflicting results in the literature. Some studies indicated that the lack of NGF did not influence pathogenesis of AD. It was found that NGF mRNA and protein levels were similar in both normal and AD brain tissue [126,127]. Therefore, pro-NGF processing and NGF retrograde transport could be a reasonable alternative to explain why NGF signaling is dysregulated in AD. In the normal brain, mature NGF (mNGF) binds to TrkA receptors on the distal ends of neurons [128]. The receptor–ligand complex is then taken up by the cell and is retrogradely transported to the neuron cell body [128]. In AD, less mNGF is produced and taken up by cholinergic neurons. Studies have demonstrated that pro-NGF is the principal form of NGF present in AD brains [129,130]. The increased levels of pro-NGF present in AD brains are due to the lack of processing of pro-NGF to mNGF as well as the increase in degradation of mNGF. In the extracellular space, pro-NGF is cleaved into mNGF by the plasmin enzyme [131,132]. These enzymes are released upon the release of pro-NGF from neurons [132]. Matrix metalloproteinase 9 (MMP-9) degrades mNGF to remove it from the system, and MMP-9 has a higher level in AD patients compared to healthy individuals [132,133]. It was found that AD patients had a higher serum level of plasmin activator inhibitor 1; the low plasmin levels contribute to the lowered amount of mNGF [134,135]. Chronic inhibition of pro-NGF to mNGF maturation leads to cholinergic neuron atrophy and loss of p75^NTR^ and TrkA receptors [136]. In addition, inhibition of pro-NGF processing led to inhibition of consolidating recent memories and reduced cholinergic presynaptic bouton density in mice [131]. Altering the balance of pro-NGF and mNGF to favor pro-NGF in a transgenic mouse model led to a deficit in memory and learning along with an increase in APP processing to amyloid beta [137]. In addition, inhibiting the degradation of mNGF by MMP9 inhibitors led to an increase in cholinergic presynaptic bouton density [131]. All of these points suggest that the accumulation of pro-NGF and the degradation of mNGF lead to problems in cholinergic neurons. Therefore, levels of mNGF are lower in AD brains, leading to the worsening of cholinergic neuron existence.

Alternatively, pro-NGF has been shown by other investigators to have similar activity to mNGF. Pro-NGF still activates TrkA and promotes neurite outgrowth [138]. Pro-NGF that is resistant to cleavage into mNGF can support the survival of neurons in cell culture [139]. In contrast, the cleavage-resistant pro-NGF has less activity than the parent pro-NGF for the phosphorylation of TrkA [139]. The relative levels of TrkA and p75^NTR^ could be important in determining the activity of pro-NGF [138,140]. When TrkA was knocked out, pro-NGF caused cell death in PC12 cells, and this effect was not observed with mNGF [140]. It is still not clear whether pro-NGF generates the observed neurodegeneration in AD or the imbalance of NGF receptors causes neurodegeneration.

The delivery of NGF to the brain has been attempted to help treat various neurodegenerative diseases. Due to its physicochemical properties, NGF has difficulty in passively penetrating the BBB [141]. Indeed, many clinical trials focusing on the systemic delivery of NGF have failed either due to lack of efficacy or the presence of pain as a side effect [142]. To overcome the problem with systemic delivery, more direct brain delivery approaches have been attempted, such as ICV infusion of NGF, which, in one study, led to increased memory retention in aged rats [143]. Rats infused with NGF via ICV had better performance in a water maze spatial memory test and had higher choline acetyltransferase activity in the basal forebrain in an ibotenic acid lesion model [144]. ICV infusion of NGF was able to prevent cholinergic neuron degeneration after lesion induction through fimbria–fornix transection in non-human primates [145]. Unfortunately, these studies had issues with side effects such as an increase in pain sensitivity in rats [144,146]. These studies found that ICV infusion of NGF led to weight loss in rats [144,146]. A small clinical trial involving an ICV infusion of NGF in AD patients showed improvements in a subset of the cognitive tests; however, NGF also caused pain and loss of appetite and weight [147]. Overall, ICV infusion of NGF has limitations for treatment of AD due to adverse side effects.

As an alternative to ICV Infusion, NGF was administered via intraparenchymal injection in which NGF was injected directly to the treatment area, such as the basal forebrain in AD [144,146,148,149]. In preclinical models, intraparenchymal injection allows NGF to reach the site of action without the side effects associated with ICV infusion [144,146]. Studies have shown that intraparenchymal injection of NGF effectively activates and sustains phosphorylation of TrkA receptor [148]. Intraparenchymal injection of NGF in rats prevented cholinergic neurodegeneration and improved the performance of rats in a water maze after ibotenic acid lesions in the basal forebrain [144,149]. Most notably, the intraparenchymal administration of NGF did not produce the adverse side effects associated with ICV administration of NGF [144,146]. Overall, intraparenchymal administration seems to be advantageous relative to ICV administration in which NGF retains its efficacy without showing adverse side effects. However, intraparenchymal injections still have the disadvantage of being an invasive procedure with risk of infection.

#### NGF Intranasal Delivery in AD and Other Brain Diseases

Multiple studies have used the IN route to deliver NGF to the brain of AD mouse models [150,151,152]. NGF delivered through the IN route reduced amyloid β plaques in the APP/PS1 and AD11 mouse models of AD [152,153]. IN-administered NGF also prevented the decline in visual recognition and spatial memory in AD11 mice [154]. IN administration of NGF^P61S/R100E^ or the P61S/R100E mutant of NGF in the 5XFAD mouse model improved spatial memory performance as well as reduced the pain side effect of NGF [150]. The IN-administered NGF^P61S/R100E^ was able to reduce the amount of amyloid plaques in the 5XFAD mice [150]. Similar neurocognitive effects were seen in other AD mouse models when NGF^P61S/R100E^ was given through the IN route, with similar activity to that of native NGF [151]. The effects of NGF^P61S/R100E^ were due to signaling in the microglia and astrocytes that led to the decrease in the release of tumor necrosis factor alpha, with a subsequent upregulation of the chemokine CXCL12 in the neurons [150].

NGF administered via IN was investigated for treating spinal cord and brain injuries [155]. In a mouse model for traumatic brain injury, administration of NGF via IN promoted a faster recovery in injured mice compare to those untreated injured mice [155]. A similar result was observed in a rat model of traumatic brain injury [156]. IN-delivered NGF was able to reduce neuroinflammation at the brain injury and distant sites [156]. Epileptic seizure duration can be shortened upon administration of NGF via IN by promoting neuronal survival; it also decreased the expression of p75^NTR^ receptor along with the activation of the Caspace-3 pathway [157].

### 6.2. NGF in Multiple Sclerosis, Parkinson’s Disease, and Stroke

Similar to BDNF, NGF also has a role in the development of MS. NGF deprivation leads to the upregulation of microRNA-219, which can cause an increase in the differentiation of oligodendrocyte precursor cells to oligodendrocytes in vitro [158]. NGF treatment led to an increase in axon myelination in various brain regions in the AD11 mouse model [158]. In contrast, similar studies showed that NGF treatments prevent oligodendrocytes from myelinating dorsal root ganglion cells [159]. Interestingly, TrkA activation in neurons by NGF prevented their myelination [159]. No expression of TrkA and p75^NTR^ in oligodendrocytes was observed within MS lesions [160]. In addition, reactive astrocytes on the edge of MS lesions were found to express p75^NTR^ [160]. In vitro, NGF activation of p75^NTR^ caused oligodendrocyte cell death [161]. All of the previous results suggest that mature oligodendrocytes are not affected by NGF, or negative effects can occur when treated with NGF. NGF may affect oligodendrocyte progenitor cells (OPC), which are important for re-myelinating neurons. OPC initially expresses high levels of TrkA after differentiation from neural stem cells, and NGF is required to efficiently differentiate OPCs into mature oligodendrocytes [162]. Given that NGF could potentially negatively affect oligodendrocytes, more studies will need to be carried out to determine the suitability of NGF as a treatment for MS.

NGF may have a limited use in the treatment of PD because the substantia nigra does not express TrkA receptors [86]. Nevertheless, NGF administration reduced circling behavior in a 6-hydroxydopamine (6-OHDA) mouse model of PD after amphetamine induction [163]. In a single individual clinical trial for PD, NGF was administered after adrenal chromaffin autograft at the putamen to help the cell graft survive [164]. Perhaps the administration of NGF could affect other neuron populations but not in the substantia nigra, where it is needed. Therefore, more studies are needed to determine if NGF would be a suitable treatment for PD.

The NGF gene or mRNA can also be delivered to the brain as an alternative to protein as therapeutics [165,166,167]. Relative to proteins, mRNA can be produced relatively easier, and cells can make the correct post-translational modifications to the protein once the mRNA has been delivered. Exosomes generated from human embryonic kidney (HEK) 293 cells were used to deliver NGF mRNA to the brain [165]. To help target the brain, rabies virus glycoprotein was fused with the lysosome-associated membrane protein 2b on the exterior of the exosome [165]. The exosomes loaded with NGF mRNA were administered intravenously to promote neurogenesis and prevent cell death in an ischemic brain injury mouse model [165]. Similarly, treatment of peripheral neuropathy was successful using NGF mRNA-loaded lipid nanoparticles (LNP) [166]. NGF^R100W^, a mutant form of NGF, can activate the neuroprotective pathways with a lower pain sensitivity side effect after administration [166]. LNPs loaded with NGF^R100W^ mRNA can ameliorate peripheral neuropathy after local delivery to the site of damage due to chemotherapy [166]. The main disadvantage was that i.v. administration led to accumulation of the lipid nanoparticles to the liver [166]. Therefore, this rapid clearance of LNP could limit the potential usefulness of nanoparticles for delivering NGF to the CNS. In addition, without help from other means of opening the BBB or enhanced uptake through endocytosis process, the efficiency of delivering LNP to the brain may be low. Liposomes studded with transferrin ligand and cell-penetrating peptide (CCP) were used to deliver an NGF plasmid DNA to the brain. These liposomes reduced the soluble and insoluble amyloid beta in the APP/PS1 mouse model of AD [167]. Overall, NGF does have some utility in treating some nervous system injuries and diseases. However, NGF possesses a limited utility in the treatment of neurodegenerative diseases such as MS and PD. Finding the most efficient delivery method will also be essential for NGF to become a treatment for neurodegenerative diseases.

## 7. Insulin-like Growth Factor-1 (IGF-1) in Neurodegenerative Brain Diseases and Brain Injury

**Figure 2:**
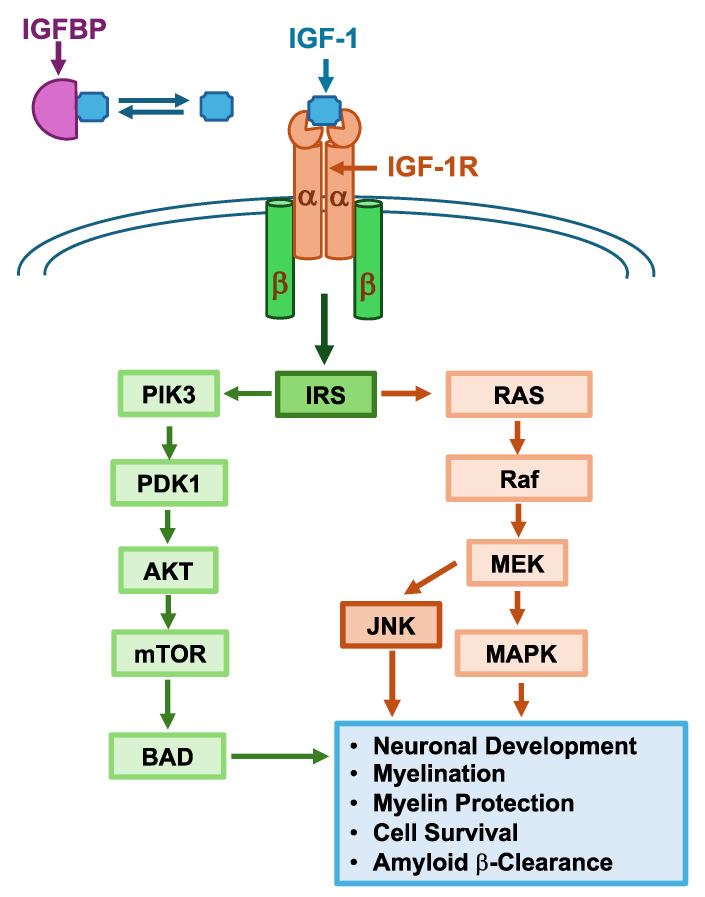
The signaling mechanism of IGF-1 upon binding to IGF-1 receptor (IGF-1R). In the extracellular, IGF-1 interacts with IGF binding protein (IGFBP). IGF-1R is a homodimeric receptor, and when activated, it has cellular effects in neuroregeneration or neuro-repair. Upon IGF-1 binding to IGF-1R, tyrosine residues on IGF-1R are autophosphorylated, creating binding sites for substrates such as insulin receptor substrate (IRS). IRS can bind to the receptor, allowing multiple pathways to be activated. In the first pathway, PIK3 can bind to IRS, which can activate AKT, ultimately inhibiting BAD and resulting in the cell survival and other beneficial effects. In the second pathway, the MAPK pathway is activated starting with the activation of Ras. The effects of activating this pathway are similar to activation of the PIK3 pathway. These signaling events ultimately lead to cell survival, myelination and myelin protection, and neuronal development. Abbreviations: IGF-1, insulin-like growth factor 1; IGFBP, insulin-like growth factor binding protein; IGF-1R, insulin-like growth factor 1 receptor; IRS, insulin receptor substrate; PI3K, phosphatidylinositol 3-kinase; PDK1, 3′-phosphoinosotide-dependent kinase-1; AKT, protein kinase B; mTOR, mammalian target of rapamycin; BAD, BCL2-associated agonist of cell death; Ras, rat sarcoma; Raf, rapidly accelerated fibrosarcoma; MEK, mitogen-activated protein kinase kinase; MAPK, mitogen-activated protein kinase; JNK, Jun N-terminal kinase.

Insulin-like Growth Factor-1 (IGF-1) is another neurotrophic factor with important functions in the brain [168]. IGF-1 is a 70-amino-acid protein with structural similarity to insulin that is produced in both the brain and peripheral tissues [169]. The brain production of IGF-1 peaks during the perinatal period and decreases in adulthood [170]. To exert its downstream effects, IGF-1 binds to the IGF-1 receptor (IGF-1R) to engage with multiple signaling pathways, including the PIK3-AKT and Ras-mitogen activated protein kinase pathways (Figure 2) [169]. Upon IGF-1 binding to the IGF-1R, IGF-1R autophosphorylates tyrosine residues, creating docking sites for proteins such as the insulin receptor substrate (IRS) [171]. IRS can then activate various enzymes such as PIK3 or Ras, leading to activation of the PIK3/AKT and the Ras/MAPK pathways, respectively (Figure 2) [171]. For an excellent review on the IGF-1 signaling pathway, we refer the readers to Werner (2023) [171]. These pathways ultimately lead to changes in resistance to oxidative stress, resistance to apoptosis, and protein synthesis [169].

IGF-1R is a natural brain delivery system for IGF-1, and it has been utilized to deliver therapeutic proteins to the brain [33,169,172]. Besides IGF-1, insulin also binds to the IGF-1R, and IGF-1 can also bind to the insulin receptor (IR) due to promiscuity in their binding properties [169,173]. The IR and the IGF-1R can also hybridize to produce a hybrid receptor that has different affinities for IGF-1 depending on the isoform of the IR that is used in the hybridization [174]. Nevertheless, binding of insulin or IGF-1 to the receptor produces similar downstream effects in neurons [169]. It should be noted that IR activation does not lead to glucose regulation in neurons compared to other cells [169]. IGF-1 signaling is modulated by IGF binding proteins (IGFBP), and IGFBPs can either enhance or inhibit the activity of IGF-1 [175]. For example, IGF-1 activity can be reduced upon binding to IGFBP-2, while its activity can be enhanced upon binding to IGFBP-1 [175]. IGF-1Rs are expressed in both the choroid plexus and the endothelial cells of the BBB, allowing IGF-1 to enter the brain from the periphery [169,172]. Opposite to the expression of IGF-1, the IGF-1R is expressed throughout the brain and has relatively steady expression levels starting from development [176]. Therefore, peripheral IGF-1 continues to have important roles in the adult brain; however, the roles of IGF-1 produced in the brain are different than that in the peripheral [169] IGF-1 produced in the brain regulates neuronal plasticity and acts as a pro-survival factor; in contrast, peripheral IGF-1 relays information on glucose metabolism and effects the permeability of the BBB [169].

Some studies have reported that subcutaneous administration of IGF-1 has a beneficial effect in neurodegenerative conditions [177]. Nevertheless, various strategies have been used to improve brain levels of IGF-1 in brain diseases. Infantile neuronal ceroid lipofuscinosis disease mice treated with IGF-1 loaded onto mesoporous silicon nanoparticles showed a more consistent IGF-1 level compared to those administered free IGF-1 [178]. ICV administrations of IGF-1 promote motor recovery and memory after traumatic brain injury in mice [179,180,181]. Similarly, ICV-infused IGF-1 was able to reduce infarction rates and neuron loss in a rat stroke model [180]. In a similar stroke model, the neuroprotective effect of IGF1 was only observed in the hippocampus [181]. The IGF-1 gene in an adenoviral vector delivered to the brain of aged rats improved motor performance [182]. Similarly, an adenoviral vector containing the IGF-1 gene administered to the brain of traumatic brain injury in rats reduced oxidative stress markers and improved working memory [183]. Adenoviral vectors encoding the IGF-1 gene administered before induction of a stroke promoted rapid recovery in mice compared to untreated mice [184]. IN of IGF-1 improved motor function after in rats with stroke [185]. Similarly, IN administration of IGF-1 reduced brain damage from hypoxia-ischemia in newborn rats; however, the therapeutic window for the treatment was limited to 2 h post hypoxia-ischemia [186]. Similarly, the timing of IN dosing of IGF-1 is important for the efficacy in various forms of traumatic brain injuries.

### 7.1. IGF-1 in Alzheimer’s Disease

The role of IGF-1 in AD is still unclear because there are controversial findings on the roles of IGF-1 and IGF-1R. The cellular location of IGF-1R could be an important factor in the progression of AD; elevated IGF-1R is found in AD mice on astrocytes but not on neurons [187]. IGF-1-regulated astrocytic functions and overexpression of IGF-1 was shown to protect the brain from injury in mice [188]. Astrocytes may overexpress IGF-1R to compensate for lower serum IGF-1 levels in older individuals with AD. The reduced IGF-1 signaling may prevent the astrocytes from effectively clearing amyloid beta from the brain.

Various studies using different model systems have demonstrated that either reducing IGF-1R signaling or reducing serum IGF-1 can reduce AD symptoms. AD mice with inducible knockout of neuronal IGF-1R had less amyloid beta and less brain inflammation [189]. The reduction in IGF-1 signaling resulted in improved learning behavior in a Y-maze assay [189]. Knocking out the IGF-1R reduced the severity of AD symptoms and prevented AD onset in animal models [190,191,192]. AD mice that had only one copy of the IGF-1R had better cognitive ability in a Y-maze and motor skills than those of untreated AD mice [191]. The IGF-1R mutant mice also had reduced inflammation in the hippocampus and cortex compared to regular AD mice [191]. IGF-1R knockout in AD mice with pathological symptoms did not affect the maze navigation capabilities, disease progression, or amyloid β clearance; however, it protected against amyloid β oligomer proteotoxic insults [190]. Inhibition of binding between IGF-1 and IGF-1R by picropodophyllin in APP/PS1 AD mice lowered the amount of insoluble amyloid beta 1–40 based on ELISA, suggesting that reduction in IGF-1 signaling improves AD; however, immunohistochemistry did not show a large change in amyloid beta deposits [192]. Unfortunately, the AD mice were not subjected to cognitive test in this study [192]. Reducing protein intake was suggested to increase learning and memory in AD mice; this was suggested due to the lowering of serum IGF-1 [193]. This study also suggested that reducing IGF-1 signaling had a protective effect in AD, which is in contrast with previous studies, where knockout of IGF-1R led to amelioration of AD symptoms in mice.

Some studies have suggested that IGF-1 signaling helps in reducing symptoms of AD, and it is needed to prevent AD. In one study, a correlation was found between lower serum levels of IGF-1 and increased risk for AD [194]. IGF-1 signaling has beneficial effects in AD animals, and IGF-1 carotid administration led to lower amyloid beta levels in rats [195]. The beneficial effects were due to the increased clearance of amyloid β through the blood–CSF barrier; however, these results have not been replicated in other animal models [195,196]. Studies of human samples indicated that the amount of serum IGF-1 did not correlate with an increased or decreased risk of AD [197]. Similarly, the natural genetic variations of IGF-1 and IGFBP3 were not associated with the increased risk of AD [198]. These studies would suggest that IGF-1 signaling is either beneficial or has no effect on AD.

The subcellular localization of IGF-1R may be critical in AD disease progression. NFTs caused IGF-1R to move from the plasma membrane to the intracellular compartment in an AD mouse model [187]. Increased IGF-1R levels were found near NFTs in astrocytes but not in normal neurons [187]. In addition, lower levels of insulin receptor substrate-1 (IRS1) and IRS2 were found in AD neurons; IRS1 and IRS2 are involved in the signaling cascade upon IGF-1R or insulin receptor activation [187]. These findings contradict the concept that reducing IGF-1R signaling or IGF-1-level reduces AD symptoms. Insulin resistance may also play a role in the effect of IGF-1 in AD, where insulin resistance is a risk factor for developing AD.

### 7.2. The Role of IGF-1 Signaling in Parkinson’s Disease (PD)

The IGF-1 level in the midbrain is normally three times higher than in the rest of the brain; therefore, IGF-1 plays an important role, especially in dopaminergic neurons [199]. Although serum IGF-1 levels were not statistically different between PD and control groups in a clinical study, higher serum IGF-1 levels correlated with patients in the early stage of PD [200]. Early-stage PD patients, who have the highest quartile of IGF-1 serum levels, exhibited the worst PD symptoms [201]. Other studies found that serum IGF-1 levels were not different between control and PD patients [202].

Studies in cell culture and animal models have demonstrated a protective effect of IGF-1 in PD. Many of the cell culture and animal models of PD were induced either with 1-methyl-4-phenyl-1,2,3,6-tetrahydropyridine (MPTP) or 6-OHDA [203]. In this model, MPTP causes neurotoxicity to dopaminergic neurons upon its conversion to 1-methyl-4-phenylpyridiniumion (MPP^+^) in astrocytes following uptake by the dopamine transporter [203]. MPP^+^ binds to complex I in the electron transport chain, resulting in less ATP production [203]. 6-OHDA is transported into dopaminergic neurons and generates reactive oxygen species, leading to cytotoxicity [203]. In the PC12 cell line model of PD, the administered IGF-1 protected cells from toxicity induced by MPP^+^ [204]. IGF-1 can protect against excessive autophagy in the human neuroblastoma SH-SY5Y cell line exposed to MPTP [205]. In a PD mouse model exposed to MPTP, pretreatment of IGF-1 at the lateral cerebral ventricle rescued these mice from movement disorder and prevented dopaminergic neuron loss [205]. Importantly, this effect could be reversed by treatment with IGF-1R antagonists [205]. Similar results were observed in both mouse and rat PD models treated with 6-OHDA [206,207]. The regenerative effects of IGF-1 were observed weeks after lesion induction by 6-OHDA [208]. Mice heterozygous for the IGF-1R were also more vulnerable to MPTP-induced dopaminergic neuron loss [209]. These studies suggest that pretreatment with IGF-1 has protective effects in PD cells and animal models.

There is a dichotomy between what was observed in human studies and what was observed in animal studies. Clinical measurements of IGF-1 would suggest that higher levels of IGF-1 are detrimental in PD patients [200,201]. On the other hand, animal and cell culture studies demonstrated a protective effect of IGF-1 in preventing PD [210]. The IGF-1 level is potentially increased during the onset of PD; however, the methods to produce PD in animal models did not represent how humans develop PD [210]. This could explain the opposing effects of IGF-1 observed in clinical studies and animal models. In addition, serum IGF-1 levels may not be representative of how IGF-1 acts locally in the brain. As mentioned previously, systemically produced IGF-1 has different effects than locally produced IGF-1 in the brain; therefore, IGF-1 may need to be directly administered to the brain rather than in the systemic circulation to produce beneficial effects in PD.

### 7.3. IGF-1 in Multiple Sclerosis (MS)

The roles of IGF-1 in the progress of MS have been investigated, and cells involved in MS progression are affected by IGF-1 [177,211,212]. Although IGF-1 levels decrease throughout adult life, IGF-1R is consistently expressed throughout the brain in all major cell types [176]. In the context of MS, IGF-1 can increase the proliferation of oligodendrocytes to promote myelin production [213]. Serum levels of IGF-1 in MS patients were lower, but they were not significantly different than healthy adults [214,215]. In pre-clinical studies, IGF-1 and/or the IGF-1R have a role in the progression of MS [212,216]. In the EAE animal model, a model for MS, treatment with IGF-1 did not increase the disease symptoms [216]. The IGF-1R activation could stimulate CD4^+^ naïve T cells to become inflammatory Th17 cells rather than Treg cells [212].

A small clinical trial administering IGF-1 subcutaneously twice daily for 24 weeks did not yield improvements in MS as evaluated by magnetic resonance imaging [217]; however, other studies found contradictory results [177,211,218,219] Genetic removal of IGF-1R in border-associated macrophages and microglial cells led to worsened disease progression as well as increased demyelination in EAE mice [211]. Similarly, binding of a small molecule to IGF-1R reduced the histopathological hallmarks of MS in the EAE model; however, it is unknown if the small molecule activated IGF-1R or modulated the signaling pathway in some other way [219]. Continuous subcutaneous infusion of IGF-1 suppressed EAE disease symptoms when delivered before or after symptom onset [177]. IGF-1 treatment led to the proliferation of Treg cells [177]. Similarly, subcutaneous injections of IGF-1 reduced EAE symptom severity and frequency of relapses in a relapse–remitting EAE model.

Glatiramer acetate, an approved medication for MS, caused Th2 cells to secrete IGF-1 [218]. Treatment with glatiramer acetate after lysolecithin-induced spinal cord injury in animals caused remyelination in the lesion. It was suggested that IGF-1 was involved in the remyelination and mitigation of disease progression in MS [218]. IGF-1 delivery before the onset of disease symptom provided a delay in disease onset, while there was no effect when administered after the disease onset [220]. Thus, the timing of IGF-1 administration could be important for delaying onset of MS [220].

Overall, the literature is divided on the usefulness of IGF-1 in the treatment of MS. There are many studies that corroborate the idea that IGF-1 has beneficial functions on neurons and other neural cells [176,213]. Some studies have reported the beneficial effects of IGF-1 when administered to pre-clinical models of MS [177,218,219]. In contrast, multiple pre-clinical and clinical studies have found that there was little benefit in administering IGF-1 to treat MS [212,214,215,216,217]. The discrepancies in these studies may be due to differences in the dosing regimens and duration of IGF-1 administration. Some studies utilized continuous infusions of IGF-1 over various durations during disease progression, while other studies delivered IGF-1 subcutaneously. The correct dose, route of administration, duration of dose, and the effectiveness of IGF-1 entering the brain may be key factors for its beneficial effects to treat MS.

## 8. Leukemia-Inhibitory Factor (LIF) in Neurodegenerative Brain Diseases

**Figure 3:**
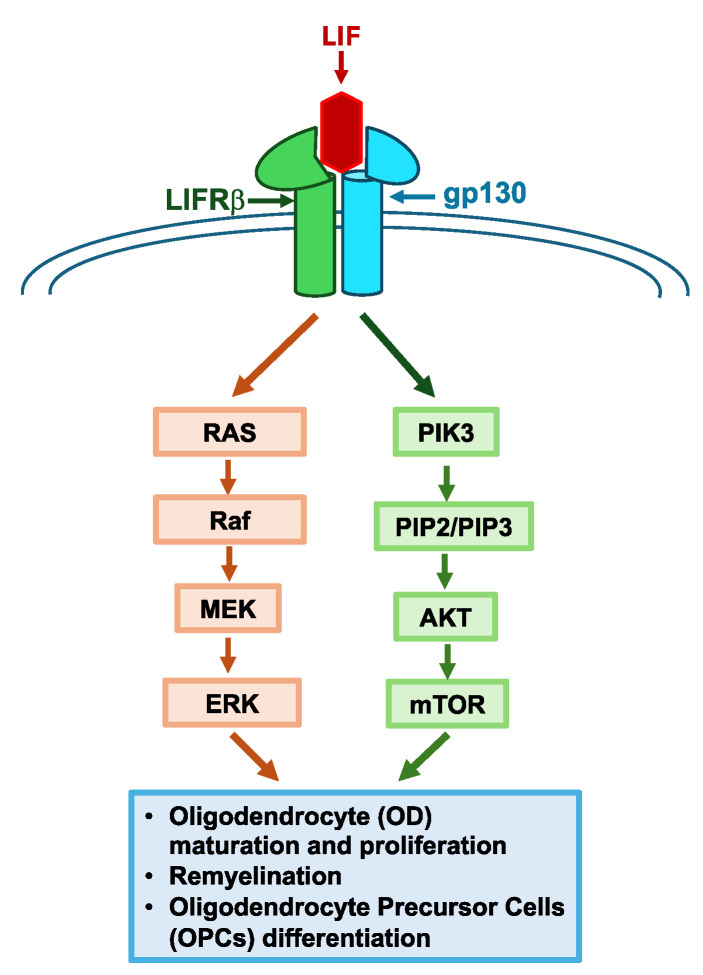
LIF signaling mechanism upon binding to LIF receptor that is constituted by LIFRβ and gp130 heterodimer. This binding process activates the RAS signaling pathway that stimulates Raf, followed by MEK and ERK signals. Another signaling process is via the PIK3 signaling process stimulated by the PIP2 and PIP3 conversion that activates AKT and mTOR signals. Both pathways produce various cellular responses, including (a) oligodendrocyte maturation and proliferation; (b) remyelination; and (c) oligodendrocyte precursor cell differentiation. Abbreviations: LIF, leukemia-inhibitory factor; LIFRβ, leukemia-inhibitory factor receptor β; gp130, glycoprotein 130; RAS, rat sarcoma; Raf, rapidly accelerated fibrosarcoma; MEK, mitogen-activated protein kinase kinase; ERK, extracellular signal regulated kinase; PI3K, phosphatidylinositol 3-kinase; PIP2/PIP3, phosphatidyl-(4,5)-bisphosphate/phosphatidylinositol-(3,4,5)-triphosphate; AKT, protein kinase B; mTOR, mammalian target of rapamycin.

Leukemia-inhibitory factor (LIF) is a cytokine that has potential in promoting neuroregeneration, and it has roles in the development of the central nervous system [221]. LIF is a part of the IL-6 family of cytokines that has a molecular weight of 20 kDa [222]; however, it has a molecular weight of 38–67 kDa in its glycosylation states [223]. LIF has three splicing variants called LIF-D, LIF-M, and LIF-T [223]. The first exon of the gene is spliced differently to produce these three variants [224]. Each variant has a different cellular location and function [224]. LIF-T is localized intracellularly, and LIF-D is localized extracellularly. LIF-M is localized both intracellularly and extracellularly [224]. The different cellular localizations of the LIF isoforms allow them to exert both autocrine and paracrine effects [223]. The LIF receptor consists of two different proteins, namely LIFRβ and gp130 (Figure 3) [222]. LIF signaling is generated when a complex of LIF and the two components of the LIF receptor is formed [222]. LIFRβ binds to Janus kinase (JAK) to induce signal transduction after assembling the signaling complex (Figure 3) [222]. JAK can then transphosphorylate another JAK, ultimately stimulating the JAK/STAT, MAPK, and PIK3 signaling pathways [222,225]. Once activated by JAK, STAT can translocate to the nucleus and alter gene expression [222]. However, STAT signaling is inhibited due to the simultaneous activation of suppressor of cytokine signaling 3 (SOCS3), which deactivates the JAK signal by ubiquitination [226]. To activate the MAPK and PIK3 signaling pathways, JAK phosphorylation results in the recruitment of SHP-2, which ultimately activates the MAPK and PIK3 pathways [222,226,227]. An excellent review on LIF signaling was published previously by Nicola and Babon (2015) [222]. Ultimately, changes in gene expression produce an increase in survival and proliferation in certain cell types.

LIF also has an important role in the development the nervous system, especially after injury. After injury to the cortex, LIF is upregulated; similarly, upregulation of LIF is observed upon injury to olfactory receptor neurons [228,229]. LIF keeps neural stem cells from differentiating and protects against the reactive oxygen species generated after stroke via upregulation of superoxide dismutase [230]. LIF promotes the survival of neural crest cells and their differentiation into sensory neurons [231]. It can also stimulate the growth of neurons from precursor cells in the spinal cord [232]. On the other hand, LIF is important in preventing neural stem cells from differentiating as well as in preventing the maturation of both olfactory neurons and cortical neurons [221,233,234]. The survival of neural stem cells is promoted by LIF, and LIF prevents their differentiation in the subventricular zone/olfactory bulb [235]. LIF causes the production of genes associated with stem cell characteristics and development [236,237]. In addition, it regulates the development and proliferation of supporting cell types such as oligodendrocytes and astrocytes [221]. Overall, LIF seems to support neural stem cells and prevent their differentiation. This fact may be important for the use of LIF as a therapeutic, where LIF could support neural stem cells to prevent them from dying.

### LIF in Multiple Sclerosis and Parkinson’s and Alzheimer’s Diseases

In neurodegenerative diseases, the role of LIF is far less characterized. LIF has been demonstrated to be useful in treating MS in animal models [221,238,239,240,241,242,243,244,245]. LIF helps proliferation of oligodendrocytes, which are important for the remyelination of axons in MS [221]. Systemic administration of LIF in EAE mice lowered the disease scores of the animals, indicating that LIF can suppress EAE [238]. More importantly, the suppression of disease scores by LIF in the EAE mice was not due to immune suppression, but it was due to increased oligodendrocyte survival [238]. Similar results were observed when an adenovirus vector (AVV) encoding the LIF gene was administered into the lateral ventricle, which caused the proliferation of oligodendrocyte progenitor cells (OPCs) and promotion of remyelination in the hippocampus [239]. Other studies have demonstrated that LIF treatment in mouse models of MS leads to remyelination [240,241]. Interestingly, LIF has been shown to downregulate the genes associated with myelination in vitro when co-administered with erythropoietin [242]. This may be due to the LIF activation of SOCS3, which leads to the inhibition of STAT3 that is responsible for axon remyelination [242]. The SOCS3-knockout mouse model showed increased myelination in the presence of LIF [243]. Unfortunately, this study only examined gene expression profile changes in the presence of LIF, without measuring degrees of myelination. In addition to the effects in oligodendrocytes and neural stem cells, LIF modulates the immune reaction associated with MS [244,245]. In MS, there is an imbalance between Treg and effector T cells such as Th-1, Th-2, and Th-17 cells. The Treg cells have the function of balancing the inflammatory T cells. Treg cells prevent inflammatory T cells from attacking the myelin sheath on the axon. In this case, LIF can promote the expression of Treg-promoting genes in CD4^+^ T cells [244,245]. Therefore, LIF may have a dual role in the suppression of MS, where both neuro-regeneration and immune tolerance are promoted by LIF.

LIF administration can also increase the number of nestin-positive neural precursor cells in a PD mouse model, which helps repair neurodegeneration in the brain [246]. Motor defects associated with PD can be alleviated by LIF [246]. In AD, LIF has benefits in protecting neurons against amyloid beta neurotoxicity [247]. On the other hand, LIF was normally found around senile plaques in AD, suggesting that it could have a role in inflammatory processes [248].

Overall, LIF seems to have promise in treating various neurodegenerative diseases. LIF’s ability to promote neuroregeneration, neural stem cell renewal, oligodendrocyte and astrocyte differentiation, and remyelination would make it useful in treating various neurodegenerative diseases. The fact that some studies have used systemic-administered LIF to treat the EAE mouse model is very promising [238,240]. LIF does seem to be able to cross the BBB through a saturable system; therefore, exogenous LIF administration may be sufficient to help in the treatment of neurodegenerative disease [249]. The role of LIF in diseases such as PD and AD needs to be better established before LIF can be considered a treatment for these diseases.

## 9. Clinical Studies and Regulatory Hurdles

Clinical trials are being performed to translate neurotrophic factors for use in humans. One current study involves using AAV vectors to deliver BDNF to AD patients and is expected to end in late 2027 [250].

Previous clinical studies with ICV-infused NGF in AD patients did not result in improvements to cognition but resulted in side effects such as weight loss and pain [147]. One clinical study involved producing autologous cells that produce NGF and then injecting them into the basal forebrain of AD patients, resulting in a reduction in the decline of mental function in AD patients [251]. Another phase 1 clinical study involving the direct injection of AAV vectors encoding NGF into the nucleus basalis of Meynert was also completed in AD patients and proved the injections did not result in any adverse effects [252]. In the following phase 2 clinical trial, injection of the AAV vector did not result in improvement in cognition relative to controls [253]. A post-mortem analysis of the phase 1 patients concluded that the AAV vectors did not spread far from the injection site and did not reach the nucleus basalis of Meynert [254]. Lack of targeting to the correct brain region may explain the lack of efficacy in the phase 2 clinical trial.

IGF-1 has also been tested in a clinical trial for the treatment of MS. IGF-1 was administered subcutaneously twice a day for 24 weeks, and MRI was used to monitor lesion size. However, no effect was noted after treatment with IGF-1 for 24 weeks [217].

Based on the clinical studies that have been performed, managing side effects will be important for the success of neurotrophic factors. Centrally infused neurotrophic factors seem to cause side effects [217]. In addition to these side effects, improving the pharmacokinetic properties of neurotrophic factors will be important for systemic administration. The short half-life of molecules would lead to the need for repeated dosing to allow sufficient levels to reach the brain [23]. Clinical trials involving clinically tested delivery methods (Table 1) will need to be carried out with neurotrophic factors to prove their ability to deliver them to the brain. The fact that some of these delivery methods have been safe when delivering other drugs is promising for their use with neurotrophic factors.

## 10. Conclusions

Treating neurodegenerative brain diseases is still very challenging because of many factors, including (a) the limited understanding of the pathology, etiology, and mechanisms of neurodegeneration of brain diseases; (b) the ability to diagnose various neurodegenerative diseases; (c) the slow development of therapeutics to halt and reverse the neurodegeneration; and (d) the difficulty in delivering therapeutic and diagnostic molecules to the CNS. One way to reverse neurodegenerative brain diseases is by delivering neurotrophic factors or molecules that can regenerate neurons. Although there are many molecules that can be used for this purpose, the brain delivery efficiency, efficacy, and safety of these molecules need further investigation. Therefore, the development of efficient brain delivery methods for neuroregenerative molecules (i.e., BDNF, NGF, IGF-1, and LIF) is essential to be able to use these molecules to reverse neurodegenerative brain diseases. The selection of molecules and target brain region(s) for neuroregeneration will be essential for the success of the treatment. The usefulness of a certain neurotrophic factor in treating neurodegenerative diseases is still uncertain because some studies contradict one another in its effectiveness. One important factor to consider is the efficiency of the brain delivery method for these therapeutic agents [24,25]. Discrepancies in the delivered doses of neurotrophic factors as well as the methods used to deliver the neurotrophic factors may be the cause of the conflicting results in the literature. For example, many previous studies utilized subcutaneous administrations of IGF-1 for treatment of neurodegenerative diseases. Although IGF-1R is present in the brain to facilitate the transport of IGF-1 to the brain, it is possible that there is not a sufficient level of IGF-1 that reaches the brain to produce the expected therapeutic effect. Therefore, an effective brain delivery method for the neurotrophic factor should be utilized to fully understand its therapeutic potential for neurodegenerative disease. Finally, another factor to consider is how to retain the neurotrophic factor in the brain for a sufficient time for it to display its activity in the brain [24,25].

## Figures and Tables

**Table 1:** Summary of mechanism and clinical uses of various blood–brain barrier-modulating methods.

Method	Mechanism	Invasiveness	Clinical Use	Citations
Intracerebroventricular injection	Direct injection into cerebrospinal fluid	Invasive	Yes, for infections and cancer	[26,28,29,30,31]
Receptor-mediated transcytosis	Molecules transport across BBB through transcytosis	Non-invasive	Approved for lysosomal storage diseases	[16,24,104,255,256,257]
Hyperosmotic mannitol	Shrinkage of endothelial cells resulting in opening of paracellular junctions	Slightly invasive	Glioblastoma	[24,36,37,38,39]
Focused ultrasound with microbubbles (FUSMB)	Ultrasound-mediated oscillation of microbubbles causes disruption of paracellular junctions	Non-invasive	Use in brain tumors	[40,41,42,43,44,62,114]
Cadherin peptides	Inhibition of cadherin–cadherin interactions in paracellular junctions resulting in opening	Non-invasive	Not tested in clinical trials	[24,25,45,46,47,48,49,50,51,52,53,54]
Intranasal	Molecules travel through paracellular pathway into the brain	Non-invasive	Clinical trials for traumatic injury with NGF	[4,55,56,57,58,59,60,61,62,63]

## Abbreviations

AAV = adeno-associated viruses; Aβ = amyloid-β; α4β1 = alpha-4 beta-1; ALS = amyotrophic lateral sclerosis; APP mice = amyloid precursor protein transgenic mice; BBB = blood–brain barrier; BBBM = blood–brain barrier modulator (BBBM); BDNF = brain-derived neurotrophic factor; AD = Alzheimer’s disease; CNS = central nervous system; CPP = cell-penetrating peptide; CREB = cAMP-response element binding protein; CSF = cerebral spinal fluid; EAE = experimental autoimmune encephalomyelitis; FUS-MB = focused ultrasound with microbubbles; HEK = human embryonic kidney; HTT = huntingtin; ICV = intracerebroventricular; IGF-1 = insulin-like growth factor 1; IR = insulin receptor; IRS = insulin receptor substrate; LIF = leukemia-inhibitory factor; LNP = lipid nanoparticles; IN = intranasal; mAb = monoclonal antibody; MPTP = 1-methyl-4-phenyl-1,2,3,6-tetrahydropyridine; MRP = multidrug-resistant proteins; MPP^+^ = 1-methyl-4-phenylpyridiniumion; MSC = mesenchymal stem cells (MSC); MS = multiple sclerosis; NGF = nerve growth factor; Nfl = neurofilament light chain; NFT = neurofibrillary tangle; NOR = novel object recognition; 6-OHDA = 6-hydroxydopamine; OPC = oligodendrocyte progenitor cells; PD = Parkinson’s disease; p75^NTR^ = p75 neurotrophin receptor; PEG = polyethylene glycol; Pgp = P-glycoprotein; PIK3 = phosphatidylinositol 3-kinase; PLC = phospholipase C-γ; PLGA = poly (lactic-co-glycolic acid); PSD-95 = postsynaptic density 95; Syn-1 = synapsin-1; PPMS = primary progressive MS; PRMS = progressive–relapsing MS; RRMS = relapsing–remitting form of MS; SPMS = secondary progressive MS; Treg = T regulatory; Trk = tropomyosin-related kinase receptors; VAMP-1 = vesicle-associated membrane protein 1.
